# Chemical Composition and Inhibitory Effect of *Lycium barbarum* L. Bud Tea and Leaf Tea on Pancreatic Lipase and *α*-Amylase Activity

**DOI:** 10.3390/foods14183167

**Published:** 2025-09-11

**Authors:** Jiayi Wei, Lutao Zhang, Jia Mi, Jiajia Wei, Qing Luo, Lu Lu, Yamei Yan

**Affiliations:** 1 School of Food Science and Engineering, Ningxia University, Yinchuan 750021, China; weijiayi0101@163.com; 2 Institute of Goji Berry Science, Ningxia Academy of Agricultural and Forestry Sciences, Yinchuan 750002, China; aquochang@foxmail.com (L.Z.); lorna0102@126.com (J.M.); weijiajia@163.com (J.W.); luoqing640603@163.com (Q.L.)

**Keywords:** *L. barbarum*, leaf tea, bud tea, widely targeted metabolomics, pancreatic lipase, *α*-Amylase

## Abstract

*Lycium barbarum* L. bud tea and leaf tea are functional processed products made from *L. barbarum* buds and leaves with traditional green tea processing techniques. Based on an extensive targeted metabolomics technology, this study systematically analyzed the chemical composition of *L. barbarum* bud tea and leaf tea, identified their differential compounds, and explored the effects of water-extracted substances on the activities of pancreatic lipase and *α*-Amylase. The results showed that the contents of total phenols, total flavonoids, and chlorogenic acid in the bud tea were 36.09 ± 1.97 mg/g, 7.44 ± 0.31 mg/g, and 4.18 ± 0.10 mg/g, respectively, 66.25%, 34.78%, and 22.58% higher than those in the leaf tea, respectively. A total of 594 metabolites were identified through the metabolomics analysis, mainly including flavonoids, phenolic acid compounds, alkaloids, amino acids and their derivatives, organic acids, lignans and coumarins, terpenoids, ands steroid compounds. Among them, flavonoids, phenolic acids, alkaloids, and amino acids and their derivatives accounted for approximately 58%. Compared with the leaf tea, the bud tea was significantly enriched with flavonoids, phenolic acid compounds, nucleotide compounds, lignans, and coumarins. Delphinidin 3-O-galactoside, cyanidin-3-glucoside, and cyanidin-3-O-glucoside were identified as significantly differential metabolites. Both *L. barbarum* bud tea and leaf tea exhibited good inhibitory effects on pancreatic lipase and *α*-Amylase, with the highest inhibition rates being 68.71%, 77.33%, 76.08%, and 69.96%, respectively. The contents of anthocyanins and their derivatives, including delphinidin-3-O-galactoside, cyanidin-3-glucoside, cyanidin-3-O-glucoside, cyanidin-O-hexoside, delphinidin-O-hexoside, and delphinidin diglucoside, were positively correlated with the activities of the two enzymes. These results underpin functional exploration and quality standardization of *L. barbarum* bud/leaf tea products.

## 1. Introduction

*Goji *(*L. barbarum*), a perennial woody plant of the genus *Lycium* in the Solanaceae family, has medicinal applications in its fruit, leaf, and root bark [1]. In Chinese historical records, *L. barbarum* buds and leaves are documented with various appellations, including “Heavenly Essence Grass”, “*L. barbarum* Tip”, and “*L. barbarum* Vegetable” [2]. These plant parts have been utilized both as food and medicine throughout Chinese history. The Supplementary Records of Famous Physicians from the Wei and Jin Dynasties [3] specify that “Roots should be collected in winter, leaves in spring and summer, while stems and fruits in autumn, followed by air-drying”. The Treatise on Medicinal Properties [4] further elaborates, “*L. barbarum* is noted for its whole-plant utility (seed and leaf). With sweet flavor and neutral nature, it tonifies essence, addresses multiple deficiency syndromes, improves complexion, whitens skin, enhances vision, calms the mind, and promotes longevity”. In the Yuan Dynasty’s Essentials of Diet and Drink by Hu Sihui [5], *L. barbarum* leaves are described as “strengthening tendons and bones, dispelling pathogenic wind, nourishing the body, alleviating consumptive fatigue, and preventing stagnation syndromes”. Seasonal harvesting guidelines specify leaf collection during spring, summer and autumn, with seed collection in winter, all suitable for prolonged dietary use. The Compendium of Materia [6] attributes multiple therapeutic effects to *L. barbarum* leaves: “Alleviating restlessness and enhancing will power, tonifying the five impairments and seven injuries, fortifying cardiac qi, eliminating heat toxins, resolving abscesses and swellings, dispelling wind pathogens, and improving visual acuity”. Contemporary research confirms that *L. barbarum* buds and leaves contain abundant bioactive compounds, including amino acids [7], polyphenols [8], and flavonoids [9]. These constituents demonstrate hypoglycemic [10], hypolipidemic [11], and antioxidant [12] activities.

*L. barbarum* bud tea and leaf tea are processed products made from the buds and leaves of leaf-used *Lycium barbarum*, utilizing traditional green tea production techniques [13]. These teas have gradually gained consumer recognition and popularity due to their rich bioactive components. Li et al. [14] reported that the iron content in Ningxia *L. barbarum* leaf tea exceeded those of oolong tea and Guanyinwang tea, while its zinc content was higher than those of Mingxiang tea and Guanyinwang tea. Long-term consumption of *L. barbarum* leaf tea can supplement essential trace elements required by the human body. Pollini et al. [15] discovered that the total flavonoid extract from *L. barbarum* leaf exhibited significant inhibitory activity against pancreatic lipase and *α*-amylase, with chlorogenic acid being the most active compound, followed by caffeic acid and salicylic acid. This suggests its potential application as a green functional food ingredient or additive for blood glucose and lipid regulation. Andrei et al. [16] found that *L. barbarum* leaf possessed adjuvant therapeutic effects for patients with hyperglycemia and hyperlipidemia. Furthermore, in European and North American markets, *L. barbarum* leaves are highly valued as functional teas or dietary supplements [17]. In recent years, research on tea’s hypoglycemic and lipid-lowering effects has gradually increased. For example, Liu et al. [18] found that green tea extract exhibited significant inhibitory activity against both pancreatic lipase and *α*-amylase. Xiao et al. [19] reported that different extracts of red honey tea possessed anti-diabetic and anti-obesity potential. Short-term and long-term consumption of Yunkang 10 green tea combined with exercise effectively alleviated fatty liver and obesity complications by alleviating hepatic inflammation, reducing lipid synthesis, and accelerating glucose transport [20]. Numerous studies have confirmed tea’s evident hypoglycemic efficacy [21], although its active components, mechanisms of action, and structure–activity relationships require further investigations. However, to our knowledge, research reports specifically addressing *L. barbarum* bud tea and leaf tea remain scarce.

With growing public health awareness, tea’s functional properties have gained increasing recognition, particularly in research fields, such as blood glucose regulation, lipid reduction, and blood pressure control, making it a hotspot in tea studies. This research used Ultra Performance Liquid Chromatography–Tandem Mass Spectrometry (UPLC-MS/MS) combined with widely targeted metabolomics technology to systematically analyze and compare metabolites in *L. barbarum* bud tea and leaf tea. This study aimed to elucidate their characteristic chemical profiles and differences and further explore the relationship between tea metabolites and the activities of pancreatic lipase and *α*-Amylase. These findings are expected to provide a theoretical basis for developing functional foods from *L. barbarum* tea products.

## 2. Materials and Methods

### 2.1. Materials and Reagents

Both *L. barbarum* buds and leaves were collected from the Germplasm Resource Nursery of the National Goji Engineering and Technology Research Center (Yinchuan, Ningxia). The harvesting and manufacturing time was in late June. Their commercial value is closely related to the maturity of fresh leaves. In the study, the harvested parts were 4.0–4.5 cm in length from the top down, which were equally divided into two parts. It should be noted that seasonal and variety differences were not evaluated in this study, which may limit the universality of the results.

The first part was subjected to hot-air fixation treatment: 100 g of fresh leaves were placed in a baking and aroma-enhancing machine, subjected to deactivation of enzymes at 120 °C for 10 min, and then taken out for spreading and cooling. Subsequently, the leaves were dried at 90 °C and then processed into *L. barbarum* bud tea (YA) and *L. barbarum* leaf tea (YE) through artificial stir-frying and other processes.

The second part was used for metabolome determination: liquid nitrogen was used for fixation, and the samples were stored in a −80 °C ultra-low temperature refrigerator for later determination. Each sampling was repeated three times. Both types of samples were uniformly subjected to vacuum freeze-drying (with a moisture content of <6%), crushed, sealed, protected from light, and stored at room temperature for subsequent use.

Reagents and suppliers: 2-Chlorophenylalanine (Merck, Germany); methanol and acetonitrile (HPLC grade, Merck, Darmstadt, Germany); porcine pancreatic lipase (Type II, 1000–2000 U/mg, Sigma-Aldrich, Portland, OR, USA); Triton X-100 (Beijing Bio-Top Biotechnology Co., Ltd., Beijing, China); *α*-Amylase (activity ≥ 10,000 U/g, Shandong XiYa Chemical Industry Co., Ltd., Linyi, China); soluble starch and phenol (Tianjin Damao Chemical Reagent Factory, Tianjin, China); p-nitrophenyl palmitate (PNPP) (Sigma-Aldrich, USA); gum arabic (analytical grade, Sigma-Aldrich, USA); sodium deoxycholate (analytical grade, Sigma-Aldrich, USA); and 3,5-dinitrosalicylic acid (DNS) (Chengdu Aikeda Chemical Reagent Co., Ltd., Chengdu, China). Additionally, sodium acetate anhydrous, sodium potassium tartrate, sodium sulfite anhydrous, sodium hydroxide, formic acid, and ethanol, all of domestic analytical grade (Tianjin Damao Chemical Reagent Factory, Tianjin, China), were also used.

### 2.2. Methods

#### 2.2.1. Extract Preparation

*L. barbarum* bud tea and leaf tea powders were mixed with preheated deionized water (96 °C, solid-to-liquid ratio 1:30) and heated in a 96 °C water bath for 30 min to obtain extracts. The mixture was centrifuged at 10,000 rpm for 6 min to yield clarified tea infusion. The tea residue underwent three repeated extractions following the same procedure, and all infusions were combined to obtain the crude extract, which was stored at 4 °C for subsequent uses [22]. It is worth noting that although this extraction condition maximizes yield, it is slightly different from the typical home brewing (this extraction time is slightly longer than the traditional brewing).

#### 2.2.2. Determination of Total Polyphenols (TPC), Total Flavonoids (TFC), and Chlorogenic Acid (CGA)

Total polyphenol content was measured according to the method described by Derakhshan et al. [22]. Total flavonoid content was determined using the protocol from Yee et al. [23]. Chlorogenic acid content was quantified following the procedure by Fetene et al. [24]. All experiments were performed in triplicate.

#### 2.2.3. Pancreatic Lipase Inhibition Assay

The inhibitory activity against pancreatic lipase was evaluated based on the method of Chen et al. [25], with slight modification for pancreatic lipase inhibition: 0.1 g of porcine pancreatic lipase was suspended in 5 mL of Tris–HCl buffer (50 mM, pH 7.2–7.4, containing 0.1% gum arabic and 0.2% sodium deoxycholate) and centrifuged at 2000 g for 10 min. In a 96-well plate, 10 μL of the sample, 30 μL of Tris–HCl buffer, and 150 μL of enzyme solution were combined and incubated at 37 °C for 20 min, followed by the addition of 10 μL of p-nitrophenyl palmitate (PNPP, 10 mM). Absorbance at 405 nm was monitored over 20 min, with all experiments repeated three times.

#### 2.2.4. *α*-Amylase Inhibition Assay

Theα-amylase inhibitory test was performed using a modified procedure of Telesphore et al. [26]. A volume of 250 μL of extract (1–300 mg/mL) was mixed with 250 μL of 0.02 M sodium phosphate buffer (pH 6.9) containing α-amylase at a concentration of 0.5 mg/mL. The mixture was preincubated at 25 °C for 10 min. Then, 250 μL of 1% starch solution in 0.02 M sodium phosphate buffer (pH 6.9) was added and incubated at 25 °C for another 10 min. The reaction was stopped by adding 500 μL of dinitrosalicylic acid (DNS). The tubes were then incubated in a water bath at 95 °C for 5 min and cooled at room temperature, followed by dilution with 5 mL distilled water. The optical density was measured at 540 nm. All experiments were repeated three times.

#### 2.2.5. UPLC-MS Analysis of Metabolites in *L. barbarum* Bud Tea and Leaf Tea

##### Sample Extraction

Samples were vacuum freeze-dried and ground into powder. A 0.10 g aliquot of the powder was mixed with 1.2 mL of 70% methanol solution and extracted for 12 h. After centrifugation (10,000× *g*, 10 min), the supernatant was collected, filtered through a 0.22 μm microporous membrane, and stored in injection vials for UPLC-MS/MS analysis.

##### Chromatographic and Mass Spectrometric Conditions

Briefly, 0.04% acetic acid in water and in acetonitrile were used as mobile phase A and B, respectively, with the following gradient elution plan: 0.00–10.00 min: linear increase of phase B from 5% to 95%; 10.00–11.00 min: 95% phase B; 11.00–11.10 min: phase B reduced to 5%; 11.10–14.00 min: equilibration at 5% phase B. The flow rate was 0.35 mL/min, and injection volume was 2 μL, with column temperature at 40 °C.

The following mass spectrometric conditions were used: Electrospray ionization (ESI), ESI temperature at 550 °C, ion spray voltage at 5500 V, curtain gas at 30 psi, high setting of collision-activated dissociation, and ion pairs monitored with optimized declustering potential and collision energy. Analyst 1.6.3 software was used to process data.

### 2.3. Statistical Analysis

All experiments were performed in triplicate. Results are expressed as mean ± standard deviation (SD). Data were analyzed by one-way ANOVA using SPSS 21.0. Heatmap and correlation analyses were performed with TBtools-II. Metabolomics data were processed using Analyst 1.6.3. Partial least squares (PLS-DA) regression analyses between pancreatic lipase/*α*-Amylase inhibitory activities (IC_50_ values) and flavonoid contents were performed using SIMCA-P 13.0.

## 3. Results

### 3.1. Contents of Total Polyphenols, Total Flavonoids, and Chlorogenic Acid in L. barbarum Bud Tea and Leaf Tea

**Figure 1:**
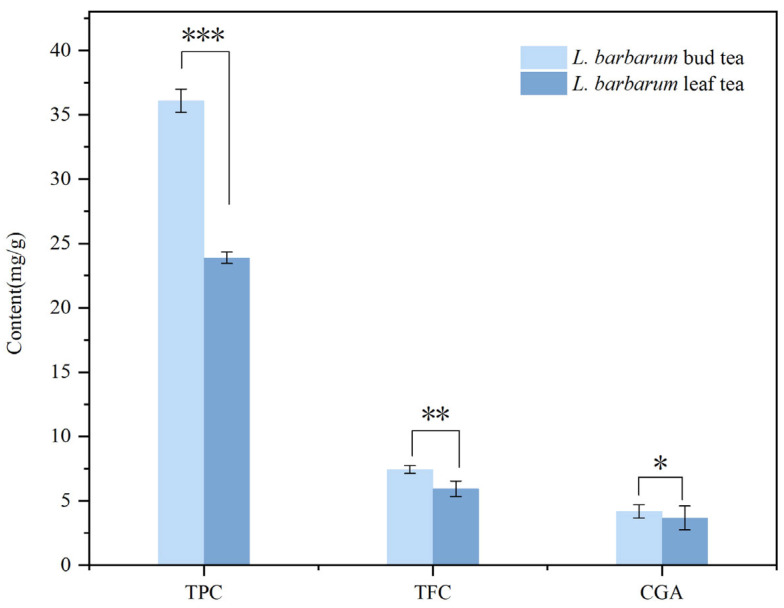
Contents of phenolic substances in *L. barbarum* bud tea and leaf tea. The results are means ± standard deviation of three replicates (*n* = 3). * denotes the significance level of the mean value between two comparison groups. * represents *p* ≤ 0.05, ** represents *p* < 0.01, and *** represents *p* < 0.001.

The total polyphenol, total flavonoid, and chlorogenic acid contents in bud tea were 36.09 ± 1.97 mg/g, 7.44 ± 0.31 mg/g, and 4.18 ± 0.10 mg/g, respectively, 1.66-, 1.35-, and 1.23-fold higher than those in leaf tea (Figure 1). Both bud tea and leaf tea exhibited significantly higher total polyphenol content relative to their total flavonoids and chlorogenic acid levels. Furthermore, the total polyphenol, flavonoid, and chlorogenic acid contents in bud tea were significantly greater than those in leaf tea processed under the same conditions, indicating that bud tea contains a richer profile of phenolic compounds compared to leaf tea. Paiva et al. [27] reported that the buds of Azorean *Camellia sinensis*, along with the first and second leaves, exhibited superior antioxidant activity and higher total phenolic content (TPC). This enhancement might be attributed to the higher concentration of polyphenols (particularly catechins) in these specific tea plant tissues. Consistent findings were reported by Rusaczonek et al. [28] who observed greater antioxidant capacity and TPC in the bud–leaf complexes of several herbal teas compared to their stem and petiole components. Furthermore, the results revealed that the combination B + 1st + 2nd L + I (buds, first leaves, second leaves with internodes) showed marginally better values across all evaluated parameters than the B + 1st + 2nd L combination (bud–leaf complex without internodes), suggesting a potential contributory role of internodal tissues in green tea quality enhancement.

### 3.2. Metabolomic Analysis of L. barbarum Bud Tea and Leaf Tea

#### 3.2.1. Identification of Components in Bud Tea and Leaf Tea

**Figure 2:**
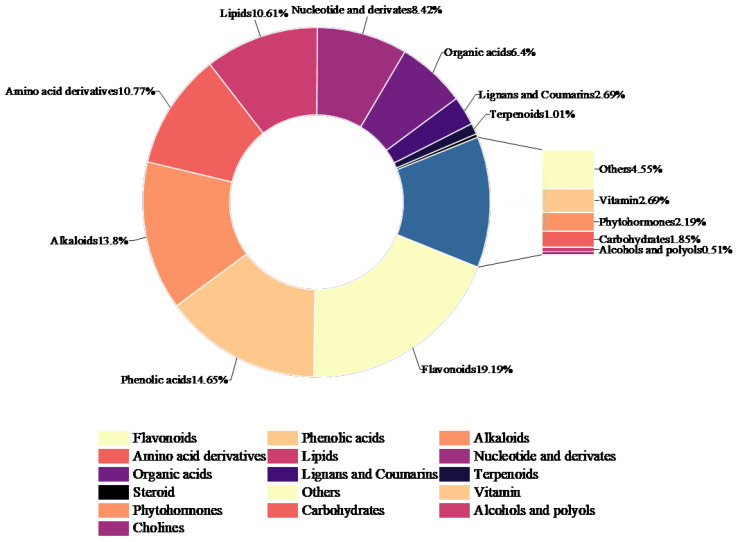
Classification of the metabolites identified in *L. barbarum* bud tea and leaf tea.

Widely targeted metabolomics analysis of *L. barbarum* bud tea and leaf tea was performed using UPLC-MS/MS (Figure 2). A total of 594 metabolites were identified and classified into 11 major categories based on their chemotaxonomic affiliations: flavonoids: 114 metabolites (19.19%); phenolic acids: 87 metabolites (14.65%); alkaloids: 82 metabolites (13.80%); others (including alcohols, cholines, sugars, vitamins, and phytohormones): 72 metabolites (12.12%); amino acids and derivatives: 64 metabolites (10.77%); organic acids: 38 metabolites (6.40%); lignans and coumarins: 16 metabolites (2.69%); terpenoids: 6 metabolites (1.01%); steroids: 2 metabolites (0.34%). Flavonoids, phenolic acids, alkaloids, and amino acids/derivatives collectively accounted for 58.41% of the total metabolites, representing the most abundant classes. The main flavonoids present in the leaves were separated and identified by high-performance liquid chromatography (HPLC), liquid chromatography–atmospheric pressure chemical ionization mass spectrometry (LC-(APCI) MS), and ultraviolet–visible spectra with shift additives [29]. The predominant flavonoid was identified as rutin.

#### 3.2.2. Clustering Heatmap Analysis

As shown in Table 1, the variance contribution rates of PC1 and PC2 are 77.113% and 14.283%, respectively. The two groups of samples show intra-group clustering and inter-group dispersion, indicating that there is a large difference between the samples and good sample repeatability.

**Figure 3:**
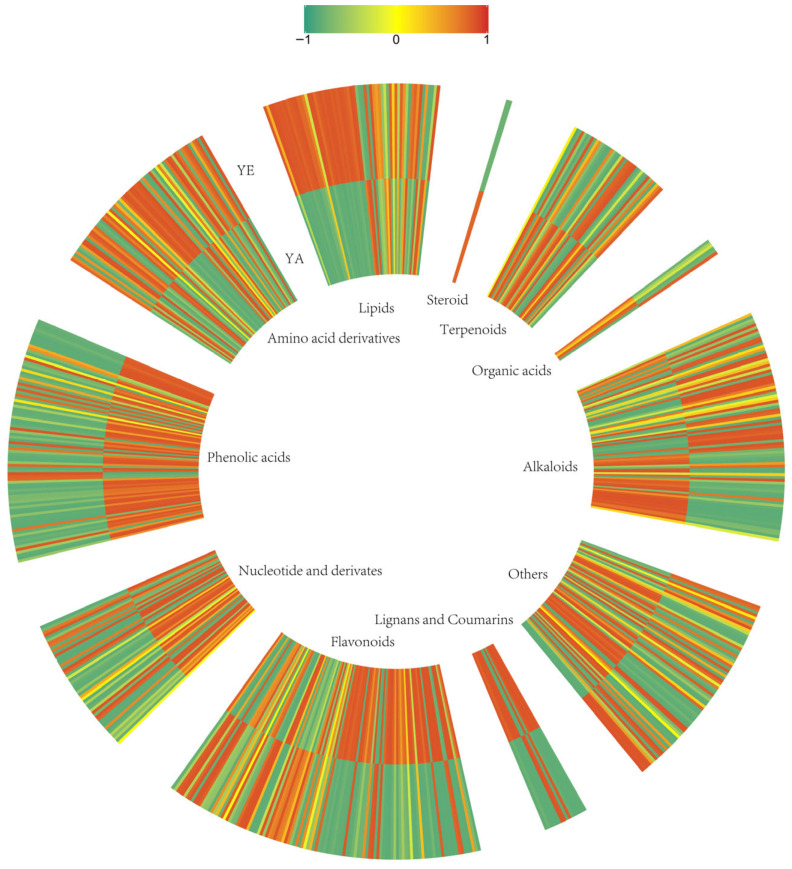
Cluster heatmap of metabolites in *L. barbarum* bud tea and leaf tea.

The clustering heatmap analysis of the metabolites in *L. barbarum* bud tea and leaf tea (Figure 3) revealed distinct compositional patterns: bud tea exhibited significant enrichment in flavonoids, phenolic acids, nucleotides, lignans and coumarins, terpenoids, steroids, and organic acids compared to leaf tea. leaf tea, in contrast, showed prominent enrichment in amino acids and derivatives, lipids and alkaloids relative to bud tea. Sun et al. [30] found that tea buds exhibited elevated expression levels of the key enzymes involved in phenylpropanoid and flavonoid biosynthetic pathways, thereby leading to higher accumulation levels of flavanols and proanthocyanidins compared to mature leaves.

#### 3.2.3. Identification and Analysis of Differential Metabolites in *L. barbarum* Bud Tea and Leaf Tea

##### Metabolite Profiling of Bud Tea and Leaf Tea

**Figure 4:**
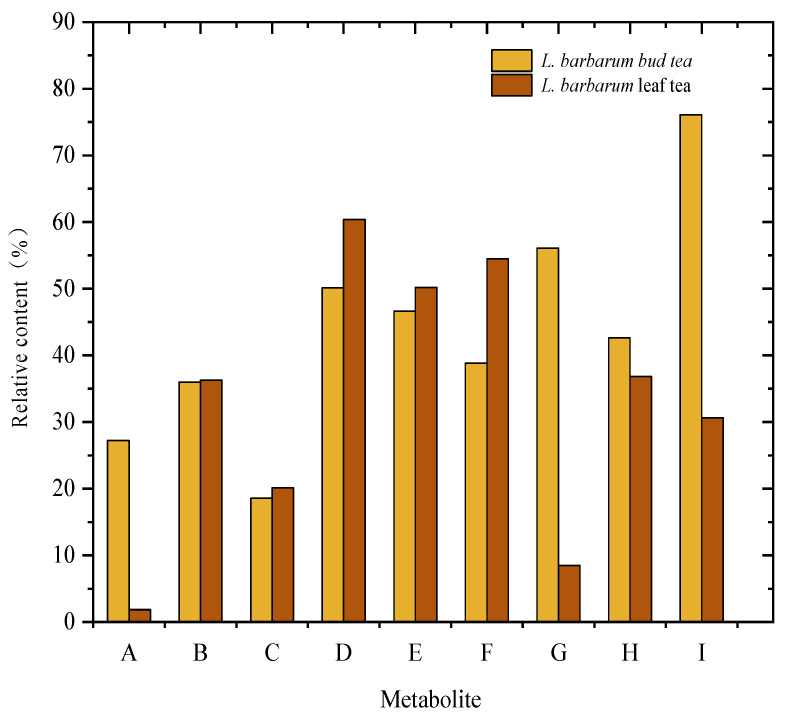
Relative contents of the same metabolite in *L. barbarum* leaf tea and bud tea Note: A: Quercetin-O-glucoside; B: Chlorogenic acid methyl ester; C: Feruloyl putrescine; D: Acetyl tryptophan; E: Lysophosphatidylcholine18:2 (2n-isomer); F: 2′-Deoxyadenosine; G: 2-Isopropylmalic acid; H: Scopoletin; I: Phytoalexin C.

The top five metabolites in relative content were screened from nine major compound categories in *L. barbarum* bud tea and leaf tea, respectively. As shown in Table 2 and Figure 4, quercetin-o-glucoside is the flavonoid compound with the highest content in bud tea, accounting for 27.22% of the total content. This bioactive flavonoid exhibited significant potential for applications in pharmaceutical and food industries [31]. It can serve as a natural antioxidant to enhance food stability and is actively studied for developing anti-inflammatory, antioxidant, and antitumor drugs [32]. Other high content metabolites, such as 2-Isopropylmalic acid, are the highest organic acid compounds in bud tea, accounting for 56.07% of the total content; Scopoletin is the lignan and coumarin compound with the highest content in bud tea, accounting for 42.64% of the total content; Phytoalexin C, the most abundant terpene compound in bud tea, constituting 76.08% of its total content. Five other metabolites show higher relative concentrations in leaf tea: chlorogenic acid methyl ester (36.13%), feruloyl putrescine (20.11%), amino acids and their derivatives (66.37%), lysophosphatidylcholine 18:2 (2n-isomer) (50.20%), and nucleotides and their derivatives (2′-deoxyadenosine) (54.48%). Notably, 2-Isopropylmalic acid is widely utilized as a pharmaceutical intermediate for drug synthesis [33] and plays a critical role in food science, particularly in flavor and aroma development [34].

##### Screening of Differential Metabolites

**Figure 5:**
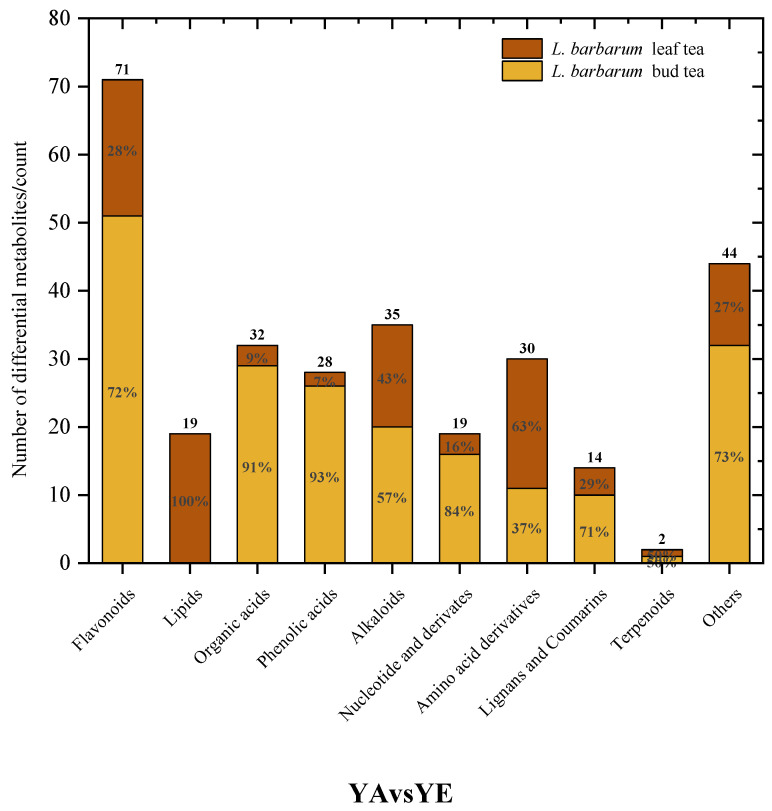
Histogram of differential metabolites classification of *L. barbarum* bud tea and leaf tea.

In order to understand the differences in the composition of metabolites in different parts of tea-used wolfberries, metabolites with a fold change ≥ 2 and a fold change ≤ 0.5 were selected. If the difference in metabolites between the experimental group and the control group is more than 2 times or less than 0.5, it is considered that the difference is significant [13]. A comparative analysis of metabolites was conducted between *L. barbarum* bud tea and *L. barbarum* leaf tea (Figure 5). There were 294 metabolites with significant differences, among which 196 substances were up-regulated, and 98 substances were down-regulated. Among them, the numbers of flavonoids, organic acids and their derivatives, phenolic acid compounds, and alkaloids in the bud tea were all higher than those in the leaf tea, while the numbers of lipids and amino acids and their derivatives in the leaf tea were higher than those in the bud tea. The results showed that *L. barbarum* bud tea contained richer phenolic substances, consistent with the result of the quantitative analysis showing that the total phenolic content in the bud tea was higher than that in the leaf tea.

The differential metabolites were sorted according to |log2FC| (Table 3), and the top 20 metabolites with the highest differential fold were identified. As shown in Table 2, among the significantly up-regulated metabolites in *L. barbarum* bud tea compared with *L. barbarum* leaf tea, 20 flavonoid compounds were screened out, which were mainly anthocyanins and anthocyanin glycosides formed by the combination of anthocyanins with sugars (such as glucose, galactose, etc.), including delphinidin-3-O-galactoside, cyanidin-3-O-glucoside, cyanidin-3-glucoside, cyanidin galactoside, delphinidin and cyanidin-3-O-glucoside. The results indicated that the main differential substances between *L. barbarum* bud tea and *L. barbarum* leaf tea were concentrated in flavonoid compounds [35]. The main flavonols in tea are flavonol glycosides, comprising mono-, di- and tri-glycosides based on quercetin, kaempferol and myricetin, and conjugated with various sugars from glucose, galactose, rhamnose, arabinose and rutinose [36,37].

The contents of EC and EGC were higher in the 1st leaf than in the bud and decreased with increasing leaf maturityv [38]. Procyanidins are important polyphenols in tea that provide many health benefits. They are made of flavanols as the structural units and polymerized by C-C bonds. In this study, the levels of procyanidin B1, procyanidin B2, procyanidin B3, and procyanidin C1 were higher in the bud than in the first, second, third, and fourth leaves. For example, the content of procyanidin B1 in the bud was 2.6-, 2.7-, 4.0-, and 5.4-fold greater than those in the first, second, third, and fourth leaves, respectively. That is, the more tender the tea is, the higher the procyanidin content [30].

### 3.3. Influence of Water Extracts of L. barbarum Bud Tea and L. barbarum Leaf Tea on the Inhibitory Rate of Pancreatic Lipase and α-Amylase

**Figure 6:**
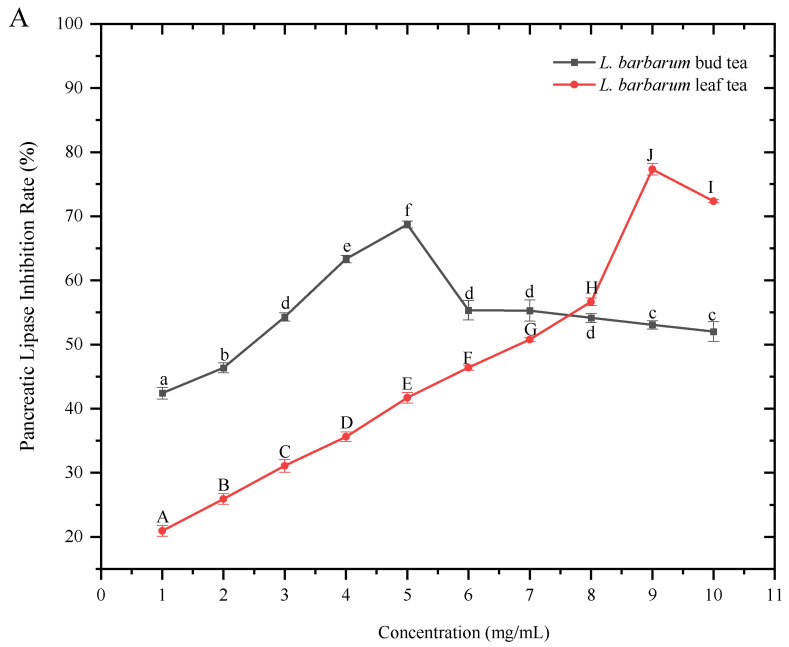

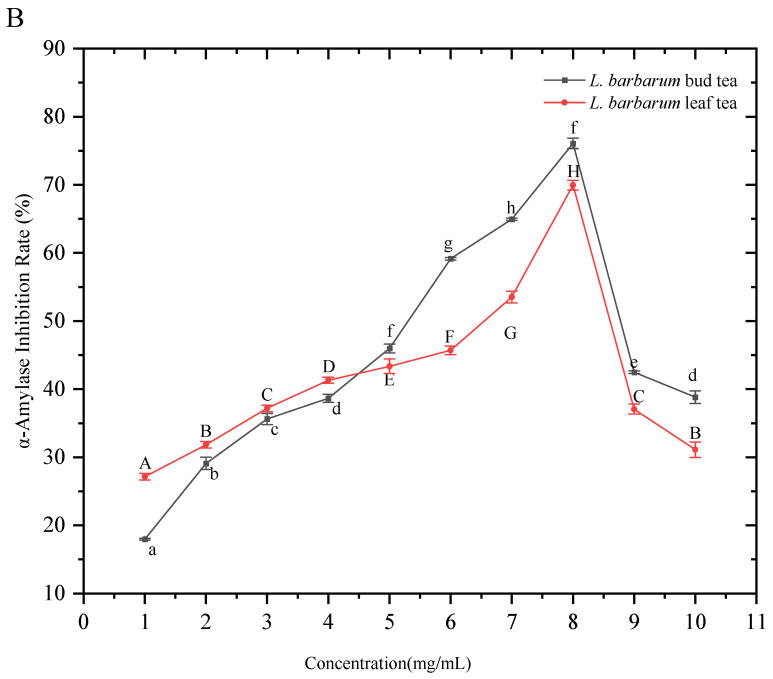
Effect of *L. barbarum* bud tea and leaf tea extract on inhibition rates of pancreatic lipase (**A**) and *α*-Amylase (**B**) The results are means ± standard deviation of three replicates (*n* = 3). The difference between different lowercase letters represents the statistical difference (*p* < 0.05) in the average inhibitory rate of pancreatic lipase and *α*-amylase of barbary *L. barbarum* bud tea, and the difference between different uppercase letters represents the statistical difference (*p* < 0.05) in the average inhibitory rate of pancreatic lipase and *α*-Amylase of *L. barbarum* leaf tea.

As shown in (Figure 6A), (Table 4)the inhibitory rate of pancreatic lipase by the extracts of *L. barbarum* bud tea and *L. barbarum* leaf tea first increased and then decreased with the increase in the extract concentration, indicating that both *L. barbarum* bud tea and leaf tea had a good inhibitory effect on pancreatic lipase. When the concentration of the tea extract was in the range of 0–7 mg/mL, the inhibitory effect of *L. barbarum* bud tea was much higher than that of *L. barbarum* leaf tea. However, when the concentration was greater than 8 mg/mL, the effect of *L. barbarum* leaf tea increased significantly and reached the highest inhibitory rate (77.33 ± 0.88%) at 9 mg/mL. While the *L. barbarum* bud tea reached the highest inhibitory rate (68.71 ± 0.54%) at 5 mg/mL, and then the inhibitory effect decreased with the increase in the concentration. This showed that the water extracts of both *L. barbarum* bud tea and leaf tea had a good inhibitory effect on pancreatic lipase.

The inhibitory rate of *α*-amylase by the extracts of *L. barbarum* bud tea and *L. barbarum* leaf tea first increased and then decreased with the increase in the extract concentration, and the inhibitory trends were basically the same (Figure 6B), indicating that both *L. barbarum* bud tea and leaf tea had a good inhibitory effect on *α*-Amylase. The *L. barbarum* bud tea reached the highest inhibitory rate of 76.08 ± 0.77% at 8 mg/mL, and the *L. barbarum* leaf tea reached the highest inhibitory rate of 69.96 ± 0.71% at the same concentration, indicating that the water extracts of both *L. barbarum* bud tea and leaf tea had a good inhibitory effect on the activity of *α*-Amylase.

It is noteworthy that “when the concentration of *L. barbarum* bud tea extract exceeds 8 mg/mL and *L. barbarum* leaf tea extract exceeds 9 mg/mL, the activity of pancreatic lipase begins to decline.” Observations on α-amylase activity revealed that “when the concentrations of both *L. barbarum* bud tea and leaf tea extracts reach 8 mg/mL, the activity of α-amylase starts to decrease. This phenomenon is caused by multiple factors. First of all, from the aggregation of compounds, it is speculated that at higher concentrations, active ingredients such as polyphenols may aggregate due to intermolecular interactions. The formed aggregates may not effectively bind to the active site of enzymes, resulting in decreased inhibitory activity. Reference analysis under similar research background. For example, SUN et al. found that: in high concentrations, tea polyphenols (e.g., EGCG) form aggregates with α-amylase, resulting in the shielding of enzyme active sites and a decrease in catalytic efficiency despite increased binding [39]. However, high concentrations of polyphenols may trigger the feedback regulation mechanism of enzymes and thus reduce the inhibitory activity. High concentrations of flavan-3-ols (e.g., catechins) form aggregates in ethanol solution through hydrogen bonding and hydrophobic interactions, resulting in reduced α-Amylase inhibitory activity. The ionic strength and ethanol content significantly affect the degree of aggregation, which is analogous to the behavior of polyphenols in high concentrations in bud tea [40]. Zhang et al. also reported that the inhibitory activity of stearic acid on α-amylase decreased with the increase in substrate (starch) concentration, which indicated that substrate competitive interference also reduced the rate of enzymatic reaction and weakened the overall inhibitory effect [41].

Pancreatic lipase, a key enzyme in dietary fat digestion and absorption, exhibits inhibitory effects that reduce lipid hydrolysis and absorption, thereby exerting anti-obesity and lipid-lowering effects. Lower IC_50_ values indicate stronger inhibitory potency. Data reveal significant differences in pancreatic lipase inhibition activity among substances: Edgeworthia gardneri tea extract (IC_50_ = 23.16 ± 0.79 μg/mL) and purple tea extract (IC_50_ = 67.4 μg/mL) demonstrated the strongest inhibitory effects [42]; *L. barbarum* bud tea (IC_50_ = 0.284 ± 0.121 mg/mL) (Table 5) ranked second, outperforming tea polyphenols (IC_50 =_ 0.41 mg/mL) and Bacillus spore-fermented green tea (IC_50_ = 0.48 mg/mL) [43]; *L. barbarum* leaf tea (IC_50_ = 0.831 ± 0.108 mg/mL) showed relatively weak inhibition, while. Chinese black tea extract (BTE) (IC_50_ = 1.016 mg/mL) exhibited the weakest effect. These variations may relate to the types and concentrations of active components [44]. Edgeworthia gardneri tea extract and purple tea likely contain high concentrations of specific polyphenols or flavonoids with strong affinity for pancreatic lipase [45], whereas *L. barbarum* bud tea demonstrates superior inhibitory efficacy compared to leaf tea due to its higher concentration of active components (e.g., polyphenols and saponins) in the bud structure.

α-Amylase is the key enzyme in carbohydrate digestion. Inhibiting its activity delays carbohydrate hydrolysis, which helps control postprandial blood glucose levels. IC_50_ Lower inhibitory values indicate stronger effects. Comparative data shows: Green tea extract (EGCG) (IC_50_ = 0.350 mg/mL) exhibits the strongest inhibition of α-amylase, followed by oolong tea polyphenols (IC_50_ = 0.375 mg/mL) and EGCG methyl derivatives (IC_50_ = 0.572 mg/mL) [46]; *L. barbarum* bud tea (IC_50_ = 0.765 ± 0.009 mg/mL) (Table 5) demonstrates better inhibitory effects than *L. barbarum* leaf tea (IC_50_ = 0.864 ± 0.113 mg/mL); Pu’er tea extracts show significant IC_50_ fluctuations (0.1–10 mg/mL) due to variations in fermentation degree and extraction methods. These differences mainly stem from active component specificity: EGCG, as the core component of tea polyphenols, may have higher affinity for α-amylase’s active site [47]; the difference between *L. barbarum* bud and leaf teas likely arises from structural adaptations of bud polyphenols and other active components to enzyme binding sites; while the IC_50_ heterogeneity in Pu’er tea confirms the conclusion that “extraction methods and material composition affect enzymatic inhibition” [48].

Overall, *L. barbarum* bud tea outperforms *L. barbarum* leaf tea in inhibiting pancreatic lipase and α-Amylase, closely related to the types, concentrations, and structural characteristics of active components. The differences among substances reflect the critical impact of component specificity on enzymatic inhibition. It should be emphasized that this experiment is a preliminary exploratory study, and no positive control was set up. Therefore, the above conclusions are still speculative and need further verification. It is particularly emphasized that the results of enzyme activity should be interpreted with caution, as the universality of the results may need to be verified by different nurseries and harvest periods.

### 3.4. Correlation of the Chemical Components in L. barbarum Bud Tea and L. barbarum Leaf Tea and Their Inhibitory Activities on Pancreatic Lipase and α-Amylase

**Figure 7:**
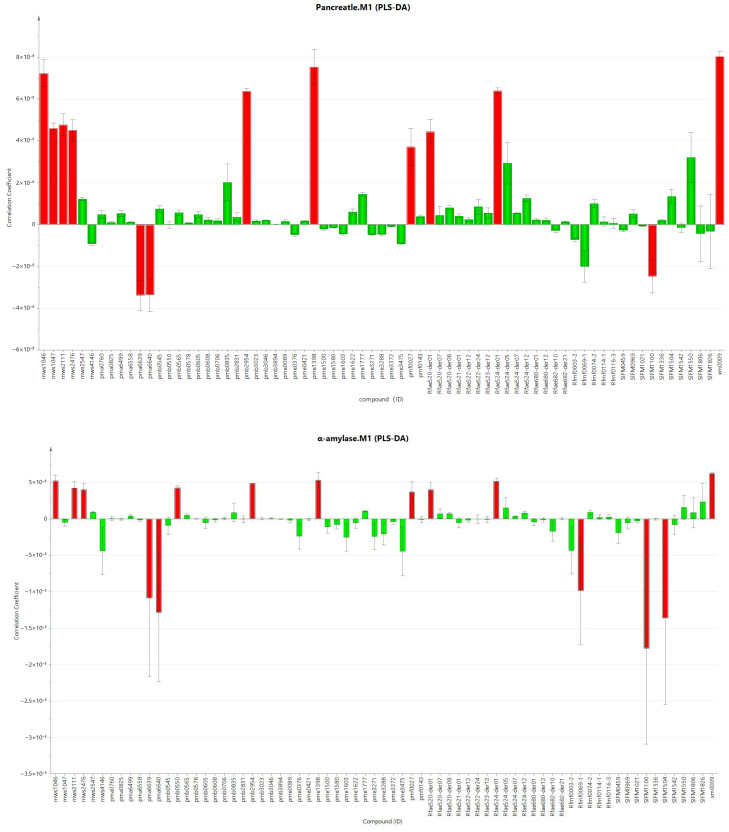
Regression coefficients of inhibition of pancreatic lipase and α-amylase activities by *L. barbarum* bud tea and leaf tea. Note: The red column represents compounds with VIP values ≥ 1, the VIP value of the compound represented by the green column is less than 1.

As shown in Figure 7, Table 6 and Table 7, among the 72 significantly different flavonoid compounds, 13 compounds with a VIP value greater than 1 and contributing to the inhibitory activity of pancreatic lipase were screened out, and there was a good correlation between the independent variable and the dependent variable (R2 = 0.987, *p* < 0.05). Among them, there were 10 bioactive compounds responsible for activity with a positive correlation, including delphinidin-3-O-galactoside, cyanidin-3-O-glucoside, cyanidin-3-glucoside, cyanidin galactoside, cyanidin-O-hexoside, delphinidin-3-O-galactoside, delphinidin-O-hexoside, delphinidin diglucoside, delphinidin-3-sophoroside-5-rhamnoside and delphinidin-3,5-diglucoside. Fifteen compounds with a VIP value greater than 1 and contributing to the inhibitory activity of *α*-amylase were screened out, and there was a good correlation between the independent variable and the dependent variable (R2 = 0.910, *p* < 0.05). Among them, there were 10 bioactive compounds responsible for activity with a positive correlation, including delphinidin-3-O-galactoside, cyanidin-3-O-glucoside, cyanidin-3-glucoside, cyanidin-3-galactoside, cyanidin-O-hexoside, delphinidin-3-O-glucoside, cyanidin-3-O-galactoside, cyanidin hexoside, delphinidin-O-hexoside and delphinidin diglucoside). There were 6 compounds that showed a significant positive correlation with the inhibitory activities of *L. barbarum* bud tea and *L. barbarum* leaf tea on pancreatic lipase and *α*-amylase (delphinidin-3-O-galactoside, cyanidin-3-glucoside, cyanidin-3-O-glucoside, cyanidin-O-hexoside, delphinidin-O-hexoside and delphinidin diglucoside). It is worth noting that in this study, the six compounds with significant positive correlation were all anthocyanins and their derivatives.

Flavonoid compounds are an important class of substances for reducing blood lipids and blood sugar [49]. Anthocyanins are a type of flavonoid compound. Due to the different substituents at the R1 and R2 carbon positions in the structure of anthocyanins, various types of anthocyanins are formed, and they rarely exist in a free state in nature, usually in the form of glycosides bound to sugars [50]. These structural features endow anthocyanins with a variety of biological activities, including antioxidant, anti-inflammatory, hypoglycemic and lipid-lowering effects. Inoue et al. [51] reported that delphinidin-3-O-galactoside mainly inhibited the generation of reactive oxygen species induced by oxidized low-density lipoprotein, and the activity and expression of nuclear transcription factor NF-κB p65. This mechanism of action indicates that this compound has antioxidant and anti-inflammatory effects. Asaki et al. [52] found that cyanidin-3-glucoside can reduce the expression of retinol-binding protein 4 (RBP4) in type 2 diabetic mice, thus improving hyperglycemia and insulin sensitivity. Cyanidin-3-O-glucoside, a typical anthocyanin pigment, was found to stimulate the activation of AMPK in HepG2 cells through CAMKK, reduce the level of malonyl-CoA, leading to enhanced fatty acid β-oxidation, and inhibition of lipid accumulation in HepG2 cells [53,54]. Liu et al. [55] reported that delphinidin-3,5-di glucoside exerts hypoglycemic and lipid-lowering effects by reducing the level of cleaved caspase-3 and increasing the phosphorylation level of AMP-activated protein kinase *α* at Thr172, thereby activating the AMPK signaling pathway to promote glucose uptake and fatty acid β-oxidation while inhibiting hepatic lipid synthesis. delphinidin-O-hexoside and cyanidin-O-hexoside exerted hypoglycemic and lipid-lowering activities by inhibiting the digestion and absorption of sugars and fats, regulating lipid metabolism, improving insulin sensitivity, and exhibited antioxidant and anti-inflammatory effects [56]. Its polysaccharide glycoside structure may affect its bioavailability, but it can still exert significant effects under the action of intestinal microorganisms.

## 4. Results and Discussion

The contents of total phenols, total flavonoids and chlorogenic acid in *L. barbarum* bud tea were 1.66, 1.35 and 1.23 times those in *L. barbarum* leaf tea. A total of 594 metabolites were identified in *L. barbarum* bud tea and *L. barbarum* leaf tea, including flavonoids, phenolic acid compounds, alkaloids, amino acids and their derivatives, organic acids, lignans and coumarins, terpenoids and steroids. Among them, flavonoids, phenolic acids, alkaloids, amino acids and their derivatives accounted for the highest proportion (58.41%). Compared with *L. barbarum* leaf tea, the components of *L. barbarum* bud tea were mainly concentrated in flavonoids, phenolic acid compounds, nucleotide compounds, lignans and coumarins, terpenoids, steroids and organic acids. Among them, delphinidin-3-O-galactoside, cyanidin-3-O-glucoside and cyanidin-3-glucoside were the significantly different metabolites between *L. barbarum* bud tea and *L. barbarum* leaf tea. Both bud tea and leaf tea exhibited good inhibitory activities on the activities of pancreatic lipase and *α*-Amylase. The 6 significantly positively correlated compounds were all anthocyanins and their derivatives, the common potential contributing components of both tea to the inhibitory activities on pancreatic lipase and *α*-Amylase. The study provides a theoretical basis for the quality evaluation of *L. barbarum* bud tea and leaf tea. While drawing these conclusions, several limitations warrant consideration. First, the study only evaluated the in vitro hypoglycemic and lipid-lowering activities of *L. barbarum* bud tea and *L. barbarum* leaf tea, without exploring their enzymatic inhibitory effects in vivo or through clinical trials, nor conducting broader tests targeting other digestive enzymes (such as α-glucosidase or cholesterol esterase). Therefore, the activity results may differ from actual in vivo pharmacological effects. Based on existing in vitro data, our findings primarily demonstrate the potential enzyme-inhibiting activity of *L. barbarum* bud tea and leaf tea extracts under specific experimental conditions, rather than directly proving their physiological functions in living organisms. It should be emphasized that this conclusion remains speculative and requires further verification. To address these limitations, we will conduct subsequent studies including cell model experiments (such as constructing disease-related cell models to validate the extract’s impact on intracellular enzyme activity and physiological indicators) and animal experiments (e.g., evaluating the extract’s metabolic kinetics, tissue distribution, and biological activity through oral administration) to comprehensively reveal its biological relevance and potential applications. Second, while the manuscript preliminarily analyzed the correlation between anthocyanins and enzyme inhibition and identified six compounds with significant positive correlations—specifically anthocyanins and their derivatives—as potential contributors to the inhibitory effects of *L. barbarum* bud tea and *L. barbarum* leaf tea on pancreatic lipase and α-amylase, the study did not isolate and characterize key anthocyanins and their derivatives for individual bioactivity testing (e.g., delphinidin-3-O-galactoside, cyanidin-3-O-glucoside). It should be noted that seasonal and variety differences were not evaluated in this study, which may limit the universality of the results.

## Figures and Tables

**Table 1:** PCA score table of metabolites in *L. barbarum* bud tea and leaf tea.

Principal Component Number	Eigenvalue	Percentage of Variance (%)	Cumulative (%)
1	458.051	77.113	77.113
2	84.842	14.283	91.396
3	51.107	8.604	100.000

**Table 2:** Relative contents of metabolites in *L. barbarum* bud tea and leaf tea.

	YA		YE	
Class	Compounds	Relative Content (%)	Compounds	Relative Content (%)
Flavonoids	Quercetin-O-glucoside	27.22	Delphinidin-3-O-glucoside	9.87
	Rutin	14.58	Delphinidin-3-O-galactoside	9.61
	Hydroxymethylflavone-7-O-hexoside	12.98	Delphinidin-O-hexoside	9.56
	Isorhamnetin-O-hexoside	10.78	Luteolin-O-hexoside	8.79
	Luteolin-7-O-glucoside	7.06	Cyanidin-3-glucoside	6.44
Phenolic acids	Chlorogenic acid methyl ester	36	Chlorogenic acid methyl ester	36.13
	Quinic acid	29.32	3-O-p-Coumaroyl quinic acid	36.08
	3-O-p-Coumaroyl quinic acid	23.8	Quinic acid	4.58
	Quinic acid-O-glucuronic acid	3.32	2,5-dihydroxy benzoic acid O-hexoside	4.16
	2,5-dihydroxy benzoic acid-O-hexoside	2.08	Quinic acid O-glucuronic acid	4.02
Alkaloids	Feruloyl putrescine	18.56	Feruloyl putrescine	20.11
	N-p-Cinnamoylagmatine	17.26	Cinnamoyl-guanidine butylamine	16.50
	Cinnamoyl-guanidine butylamine	16.51	N1,N5,N10-Tricoumaroyl spermidine	14.23
	Coumaroyl-guanidine butylamine	12.01	N-p-Cinnamoylagmatine	8.40
	Dicaffeoyl-spermidine	10.98	Coumaroyl-guanidine butylamine	6.95
Amino acid derivatives	Acetyl tryptophan	50.13	Acetyl tryptophan	66.37
	L-Pipecolic acid	19.25	5-oxoproline	9.69
	5-oxoproline	18.83	L-Pipecolic acid	9.62
	L-Asparagine	2.26	3-Hydroxy-3-methylpentane-1,5-dioic acid	2.92
	N-(3-Indolylacetyl)-L-alanine	1.77	DL-homocysteine	1.94
Lipids	Lysophosphatidylcholine18:2(2n-isomer)	46.65	Lysophosphatidylcholine18:2(2n-isomer)	50.20
	Lysophosphatidylcholine18:1(2n-isomer)	21.44	Lysophosphatidylcholine18:1(2n-isomer)	19.88
	4-Hydroxysphinganine	13.42	4-Hydroxysphinganine	14.40
	Lysophosphatidylethanolamine18:2(2n-isomer)	4.75	Lysophosphatidylethanolamine18:2(2n-isomer)	4.24
	Lysophosphatidylethanolamine16:0(2n-isomer)	2.83	Lysophosphatidylethanolamine16:0(2n-isomer)	2.97
Nucleotide and derivates	2′-Deoxyadenosine	38.85	2′-Deoxyadenosine	54.48
	Cytosine	19.07	Cyclic Guanosine-3′,5′-Monophosphate	10.33
	Adenosine-5′-monophosphate	9.94	Adenosine-5′-Monophosphate	9.6
	Guanosine	7.61	Guanosine	8.28
	Cyclic Guanosine-3′,5′-Monophosphate	6.56	Adenosine-3′, 5′-Cyclic Monophosphate	4.73
Organic acids	2-Isopropylmalic acid	56.07	Methylmalic acid	22.97
	Terephthalic acid	15.53	Terephthalic acid	16.23
	Homogentisic acid	7.20	Kynurenic acid	14.54
	Methylmalic acid	3.68	2-Isopropylmalic acid	8.47
	Kynurenic acid	3.63	2-Furoic acid	6.94
Lignans and Coumarins	Scopoletin	42.64	Scopoletin	36.86
	Esculetin	13.49	Scopolin	19.43
	Scopolin	9.77	Esculetin	17.64
	Dihydroxycoumarin-7-O-quinate	9.27	Dihydroxycoumarin-7-O-quinate	11.84
	3,4-Dihydrocoumarin	7.27	8-Methyl-2-oxo-4-phenyl-2H-chromen-7-yl 4-(hexyloxy)benzoate	5.36
Terpenoids	Phytocassane C	76.08	Roseoside	69.38
	Roseoside	23.92	Phytocassane C	30.63

**Table 3:** The number of differential metabolites in *L. barbarum* bud tea and leaf tea.

	Compounds	Class	Type	|Log2FC|
Anthocyanins and their derivatives	Delphinidin-3-O-galactoside	Flavonoids	up	10.54
	Cyanidin-3-O-glucoside	Flavonoids	up	9.87
	Cyanidin-3-glucoside	Flavonoids	up	8.99
	Cyanidin-3-galactoside	Flavonoids	up	9.06
	Delphinidin	Flavonoids	up	11.54
	Cyanidin-3-O-glucoside (Kuromanin)	Flavonoids	up	9.98
	Delphinidin-3-galactoside	Flavonoids	up	10.35
	Cyanidin galactoside	Flavonoids	up	9.20
	Cyanidin-3-O-glucoside	Flavonoids	up	8.82
	Cyanidin-hexoside	Flavonoids	up	8.77
	Cyanidin-diglucoside	Flavonoids	up	8.18
	Delphinidin-hexoside	Flavonoids	up	8.79
	Delphinidin-rutinoside	Flavonoids	up	12.65
	Delphinidin-diglucoside	Flavonoids	up	11.68
	Delphinidin-3-sophoroside-5-rhamnoside	Flavonoids	up	13.03
	Delphinidin-3, 5-diglucoside	Flavonoids	up	10.97
Flavonoids	Quercetin-3, 4′-O-di-beta-glucopyranoside	Flavonoids	up	10.03
	C-hexosyl apigenin-O-hexosyl-O-hexoside	Flavonoids	up	10.27
Catechins	Gallocatechin gallocate	Flavonoids	up	9.21
Anthocyanins	Anthocyanins	Flavonoids	up	9.66

**Table 4:** Inhibition rates of *L. barbarum* bud tea and leaf tea extract extracts on pancreatic lipase (A) and α-Amylase (B).

	Pancreatic Lipase Inhibition Rate (%)		α-Amylase Inhibition Rate (%)	
Concentration (mg/mL)	*L. barbarum* Bud Tea	*L. barbarum* Leaf Tea	*L. barbarum* Bud Tea	*L. barbarum* Leaf Tea
1	42.42 ± 0.92 a	20.95 ± 0.83 A	17.97 ± 0.14 a	27.14 ± 0.49 A
2	46.37 ± 0.78 b	25.91 ± 0.82 B	29.09 ± 0.9 b	31.83 ± 0.48 B
3	54.3 ± 0.64 d	31.09 ± 0.99 C	35.63 ± 0.82 c	37.18 ± 0.49 C
4	63.34 ± 0.59 e	35.62 ± 0.74 D	38.66 ± 0.59 d	41.31 ± 0.46 D
5	68.71 ± 0.54 f	41.69 ± 0.84 E	45.97 ± 0.63 f	43.35 ± 1.07 E
6	55.33 ± 1.52 d	46.41 ± 0.42 F	59.14 ± 0.18 g	45.7 ± 0.64 F
7	55.29 ± 1.65 d	50.77 ± 0.31 G	64.95 ± 0.17 h	53.52 ± 0.85 G
8	54.15 ± 0.69 d	56.67 ± 0.62 H	76.08 ± 0.77 f	69.96 ± 0.71 H
9	53.07 ± 0.66 c	77.33 ± 0.88 J	42.48 ± 0.21 e	37.06 ± 0.73 C
10	52.02 ± 1.53 c	72.35 ± 0.22 I	38.81 ± 0.91 d	31.11 ± 1.15 B

Note: The results are means ± standard deviation of three replicates (*n* = 3). The difference between different lowercase letters represents the statistical difference (*p* < 0.05) in the average inhibitory rate of pancreatic lipase and α-amylase of barbary *L. barbarum* bud tea, and the difference between different uppercase letters represents the statistical difference (*p* < 0.05) in the average inhibitory rate of pancreatic lipase and α-Amylase of *L. barbarum* leaf tea.

**Table 5:** The IC_50_ values of *L. barbarum* bud tea and leaf tea extracts on different enzyme activity inhibition rates.

Sample	IC_50_ (mg/mL)	
	Pancreatic Lipase	α-Amylase
*L. barbarum* bud tea	0.284 ± 0.121	0.765 ± 0.009
*L. barbarum* leaf tea	0.831 ± 0.108	0.864 ± 0.113

**Table 6:** Pancreatic Lipase-inhibiting Components in *L. barbarum* bud tea and leaf tea.

Compound (ID)	Compounds	VIP	Correlation Coefficient	Class
pma6640	Selinidin-7-O-hexoside *	1.043	−2.28 × 10^−6^	Flavonoids
SlFM1100	Quercetin-O-glucoside	1.298	−2.96 × 10^−6^	Flavonoids
SlFM1504	Rutin *	1.011	−2.38 × 10^−6^	Flavonoids
mws1046	Delphinidin-3-O-galactoside *	2.58	5.90 × 10^−6^	Flavonoids
mws2111	Cyanidin-3-glucoside *	2.107	4.81 × 10^−6^	Flavonoids
pmf0027	Cyanidin galactoside	1.997	4.22 × 10^−6^	Flavonoids
mws1047	Cyanidin-3-O-glucoside *	2.103	4.39 × 10^−6^	Flavonoids
pmb2954	Luteolin-O-hexoside *	2.464	5.63 × 10^−6^	Flavonoids
pme1398	Delphinidin-3-O-glucoside *	2.613	5.97 × 10^−6^	Flavonoids
Rfae524-der01	Delphinidin-O-hexoside *	2.573	4.82 × 10^−6^	Flavonoids
xm0009	Delphinidin-diglucoside *	2.103	2.95 × 10^−6^	Flavonoids
SlFM1826	Delphinidin-3-sophoroside-5-rhamnoside	1.118	2.55 × 10^−6^	Flavonoids
SlFM1550	Delphinidin-3, 5-diglucoside	1.128	2.87 × 10^−7^	Flavonoids

Note: * represents the repeated compounds. pma6640, SlFM1100, SlFM1504, mws1046, mws2111, pmf0027, mws1047, pmb2954, pme1398, Rfae524-der01, xm0009, SlFM1826 and SlFM1550, respectively, correspond to the following compounds Selinidin-7-O-hexoside, Quercetin-O-glucoside, Rutin, Delphinidin-3-O-galactoside, Cyanidin-3-glucoside, Cyanidin galactosideCyanidin-3-O-glucoside, Luteolin-O-hexoside, Delphinidin-3-O-glucoside, Delphinidin-O-hexoside, Delphinidin-diglucoside, Delphinidin-3-sophoroside-5-rhamnoside, Delphinidin-3, 5-diglucoside.

**Table 7:** The active ingredients in *L. barbarum* bud tea and leaf tea that inhibit α-Amylase.

Compound (ID)	Compounds	VIP	Correlation Coefficient	Class
pma6640	Selinidin-7-O-hexoside *	1.601	−1.07 × 10^−5^	Flavonoids
pma6639	Isorhamnetin-O-hexoside	1.539	−9.00 × 10^−6^	Flavonoids
SlFM1100	Quercetin-O-glucoside	1.52	−1.47 × 10^−5^	Flavonoids
SlFM1504	Rutin *	1.484	−1.13 × 10^−5^	Flavonoids
Rfmf0069-1	Malvidin-3-O-rutinoside-5-O-glucoside	1.607	−8.23 × 10^−6^	Flavonoids
mws1046	Delphinidin-3-O-galactoside *	2.839	4.91 × 10^−6^	Flavonoids
mws2111	Cyanidin-3-glucoside *	2.129	3.98 × 10^−6^	Flavonoids
mws1047	Delphinidin-3-O-galactoside *	2.092	3.64 × 10^−6^	Flavonoids
mws2476	Cyanidin-3-O-galactoside	2.065	3.77 × 10^−6^	Flavonoids
pmb2954	Luteolin-O-hexoside *	2.015	4.59 × 10^−6^	Flavonoids
pme1398	Delphinidin-3-O-glucoside *	1.971	4.97 × 10^−6^	Flavonoids
pmf0027	Cyanidin-3-O-galactoside	1.742	3.49 × 10^−6^	Flavonoids
Rfae520-der01	Cyanidin-hexoside	1.683	3.78 × 10^−6^	Flavonoids
Rfae524-der01	Delphinidin-O-hexoside *	1.681	4.85 × 10^−6^	Flavonoids
xm0009	Delphinidin-diglucoside *	1.666	2.21 × 10^−6^	Flavonoids

Note: * represents the repeated compounds. pma6640, pma6639, SlFM1100, SlFM1504, Rfmf0069-1, mws1046, mws2111, mws1047, mws2476, pmb2954, pme1398, pmf0027, Rfae520-der01, Rfae524-der01 and xm0009, respectively, correspond to the following compounds Selinidin-7-O-hexoside, Isorhamnetin-O-hexoside, Quercetin-O-glucoside, Rutin, Malvidin-3-O-rutinoside-5-O-glucoside, Delphinidin-3-O-galactoside, Cyanidin-3-glucoside, Delphinidin-3-O-galactoside, Cyanidin-3-O-galactoside, Luteolin-O-hexoside, Delphinidin-3-O-glucoside, Cyanidin-3-O-galactoside, Cyanidin-hexoside, Delphinidin-O-hexoside, Delphinidin-diglucoside.

## Data Availability

The original contributions presented in the study are included in the article, and further inquiries can be directed to the corresponding author.
